# Research protocol for a randomized controlled trial of the health effects of volunteering for seniors

**DOI:** 10.1186/s12955-015-0263-z

**Published:** 2015-06-04

**Authors:** Simone Pettigrew, Michelle Jongenelis, Robert U Newton, Jeni Warburton, Ben Jackson

**Affiliations:** School of Psychology and Speech Pathology, Curtin University, Kent St Bentley, WA 6102 Australia; School of Exercise and Health Sciences, Edith Cowan University, Perth, Australia; Faculty of Health Sciences, La Trobe University, Melbourne, VIC 3086 Australia; School of Sports Science, Exercise, and Health, University of Western Australia, Perth, Australia

**Keywords:** Volunteering, Seniors, Physical health, Psychological well-being

## Abstract

**Abstract:**

**Background:**

A growing evidence base demonstrates that interventions that focus on participation in physical and social activities can assist in preventing and treating both physical and mental health problems. In addition, there is some evidence that engaging in volunteering activities can provide beneficial social, physical, psychological, and cognitive outcomes for older people. This study will use a randomized controlled trial approach to investigate the potential for interventions involving volunteer activities to produce positive physical and psychological outcomes for older people, thereby contributing to the limited evidence relating to the potential for volunteering to provide multiple health effects.

**Methods/Design:**

This randomized controlled trial will involve 400 retired/non-employed individuals in good health aged 60+ years living in the metropolitan area in Perth, Western Australia. Participants will be recruited from the Perth metropolitan area using a variety of recruitment methods to achieve a diverse sample in terms of age, gender, and socioeconomic status. Consenting and eligible participants will be randomly assigned to an intervention (*n* = 200) or control group (*n* = 200). Those in the intervention group will be asked to engage in a minimum 60 min of volunteer activities per week for a period of 6 months, while those in the control group will be asked to maintain their existing lifestyle or take on new activities as they see fit. Physical and psychological outcomes will be assessed. Primary physical outcomes will include physical activity and sedentary time (measured using pedometers and Actigraph monitors) and physical health (measured using a battery of physical functioning tests, resting heart rate, blood pressure, BMI, and girth). Primary psychological outcomes will include psychological well-being, depression, self-esteem, and quality of life (measured using the Warwick-Edinburgh Mental Well-Being Scale, Center for Epidemiologic Studies Depression Scale, the Rosenberg Self-Esteem Survey, and the Global Quality of Life Scale, respectively). Secondary outcomes of interest will include attitudes to volunteering (measured via open-ended interviews) and personal growth, purpose in life, social support, and self-efficacy (measured using the Personal Growth and Purpose in Life subscales of Ryff’s Psychological Well-Being Scale, the Social Provisions Scale, and the Generalized Self-Efficacy Scale, respectively). Participants will be re-assessed on these measures after 6 months.

**Discussion:**

The results of this randomized controlled trial will generate new knowledge relating to the physical and psychological health benefits of different levels and types of volunteering for older people. In addition, insight will be provided into the major factors influencing the recruitment and retention of older volunteers. Understanding the full potential for volunteering to affect physical and mental well-being will provide policy makers with the evidence they require to determine appropriate investment in the volunteering sector, especially in relation to encouraging volunteering among older people who constitute an important resource for the community.

**Trial registration:**

Australian and New Zealand Clinical Trials Registry ACTRN12615000091505. Date registered: 3 February, 2015.

## Background

In Australia, consistent with global trends, there is a marked ageing of the population. Those over the age of 65 currently constitute 14.0 % of the population [1]. By 2061, more than one in five Australians will be over the age of 65, with the fastest rate of growth being among those over 85 [2].

As the population ages, the increasing incidence of age-related illnesses will have significant economic and social cost implications [3,4]. For example, older age is associated with higher body mass index scores [5,6], indicating that as the population ages, the economic and health-related costs associated with obesity will escalate. Older people also tend to engage in lower levels of physical activity than other population segments [7], and are thus in need of programs that can motivate and facilitate their engagement in higher levels of activity. As a result of their greater susceptibility to a wide range of health problems, older people have been recognized as a group requiring particular attention in the design of health promotion and illness prevention programs [8,9].

A growing evidence base demonstrates that interventions that focus on physical and social activities can assist in preventing and treating both physical and mental health problems [10-14]. There is also an emerging body of knowledge relating to sedentary time as an independent risk factor for physical illness, in particular cardiometabolic diseases [15-17]. To date, however, there appears to be no research investigating the effects of being sedentary on mental health, nor the impacts of volunteering on sedentary time. This study will explore the potential for interventions involving volunteer activities to produce positive physical and mental outcomes for older people, thereby contributing to the limited evidence relating to the potential of volunteering as a health intervention.

### Volunteering

Volunteering is defined as work activities that are unpaid, non-compulsory, and unrelated to family obligations [18]. ABS [19] data indicate that around a third of Australians engage in some form of volunteering within a 12-month period, with the highest rates among those aged 55–64 years (46 %) and 65–74 years (38 %). Previous studies suggest that volunteering has substantial health benefits for older people (for reviews see [20-22]). While causality is difficult to demonstrate due to the largely observational nature of data synthesized in reviews, evidence suggests that engaging in volunteering activities may provide beneficial social, physical, and cognitive outcomes for older people [23-32].

Volunteering has also been linked with higher levels of self-rated health, lower mortality rates, reduced risk of depression, and improved psychological well-being [20,30,33-38]. One of the mechanisms by which health benefits are produced by volunteering may be an increase in physical activity [39], which is likely to be largely due to additional manual work and walking [29]. However, there is a lack of physical evidence of this relationship, and little understanding of why it exists [31] and which forms of volunteering are most effective in generating positive health effects [40].

Motivational studies consistently demonstrate that an advantage of volunteering is that it gives older individuals an increased sense of meaning [24,41]. In a study of seniors’ conceptions of well-being [42,43], results showed that older people are interested in undertaking activities that are of benefit to others and that volunteering is salient in their deliberations about how they could achieve this outcome. However, a primary barrier was reported to be a lack of knowledge regarding volunteering opportunities and how they can be accessed. There appears to be a preference for word-of-mouth communications about volunteering opportunities, especially in the form of direct invitations to assist with specific tasks [44-46].

Volunteering is similar to physical activity in that participation rates are highest among those in better health and with higher levels of income and education [47]. It is also similar in that individuals are likely to assess the costs and benefits associated with the activity in their commencement and continuation decisions [48]. Unfortunately, little is known about how these decisions are made, and more research is needed to understand this phenomenon. Older people need particular consideration when developing health promotion messages because of their greater heterogeneity due to more extensive and varied life experiences, their stronger health-related motivations, and the physical deterioration that occurs with age that affects message processing [43,49-51]. However, little is known about the most effective ways to communicate with older people with the specific purpose of motivating them to engage in activity in general and volunteering activities in particular [52,53]. In the case of physical activity, it has been suggested that focusing on the social benefits may be more effective than emphasizing the physical health benefits [54,55]. It is likely that a similar focus on social benefits in messages aiming to encourage volunteering behaviors may be effective with the target group. As older people appear to consider mental incapacity as being more undesirable than physical incapacity [56], another approach may be to highlight the mental health benefits of participation in volunteering behaviors.

In summary, a growing body of research demonstrates the importance of combining physical, cognitive, and social activities to achieve healthy ageing. Volunteering can facilitate these activities and thus may have positive outcomes for older people. Little previous work has quantified the physical health benefits of volunteering, and even less has investigated the mental health benefits. In addition, previous studies have tended to use self-report data rather direct health measures [30]. Understanding the full potential for volunteering to affect physical and mental well-being will provide policy makers with the evidence they require to determine appropriate investment in the volunteering sector, especially in relation to encouraging volunteering among older people who constitute an important resource for the community.

The aim of this study is to assess the relative and combined effects of volunteering on seniors’ physical and mental well-being. This aim will be achieved in the context of a randomized controlled study using an Australian population sample. The primary research objective is to test whether engagement in a volunteering program results in significant improvements to physical and mental health compared to control (usual lifestyle activities). The results can be used to inform public policy on this issue and develop appropriate strategies to encourage older people to participate in volunteer activities.

## Methods/Design

The trial is funded by an ARC Discovery Grant (DP140100365) and has received ethical approval from Curtin University’s Human Research Ethics Committee (Approval reference: HR21/2014). It is a single-blind, randomized controlled trial designed to assess the impact of volunteering on seniors’ physical and mental well-being. Participants who consent will be randomized on a 1:1 basis to one of two conditions: control versus volunteering intervention.

### Sample

#### Eligibility

Table 1 summarizes the criteria used to determine participant eligibility. Potential participants must be aged 60 years or older and not have engaged in volunteering activities during the previous 12 months. Potential participants will be advised that volunteering constitutes work activities that are unpaid, non-compulsory, and unrelated to family obligations and charitable giving [18]. Those in paid employment will be excluded because their workplace participation is likely to confer physical and mental health benefits that cannot be accessed by their nonworking peers [26,57]. Non-working individuals are also likely to have the most to benefit from interventions designed to increase their physical and mental well-being due to their greater likelihood of isolation [58,59]. Potential participants will be advised that they need to be adequately mobile and physically fit to undertake the physical health tests.

**Table 1:** Inclusion and exclusion criteria

Inclusion criteria	Exclusion criteria
Fully retired	In paid employment
Age 60+	Age <60
Nil formal/regular volunteer work in last 12 months	Formal/regular volunteer work in last 12 months
Mobility and basic functional fitness	Mobility or functional fitness issues

#### Recruitment procedures

To maximize the heterogeneity of the sample, recruitment strategies will include (i) placement of notices in community newspapers and seniors’ publications, (ii) community radio announcements, (iii) distribution of flyers at seniors’ events and retirement villages, and (iv) notices distributed via local government and non-government organizations that have representation across the metropolitan area (e.g., the Council of the Aged and the Seniors Recreation Council). These varied recruitment methods will increase the likelihood of attracting a diverse sample in terms of age, gender, and socioeconomic status.

The recruitment notices will call for those aged 60+ to participate in “a study on seniors’ health”. Those who express an interest will be given details of the study and screened for their eligibility. While participants will be aware that the study relates to health among older Australians, they will be blinded to the exact nature of the research project (i.e., the existence of the intervention and control groups and the comparison of outcomes between them). Upon debriefing at the end of the study, participants will be advised of the two conditions and the full purpose of the study. At this stage, those in the control condition will be given the opportunity to consult with a volunteer agency representative should they wish to commence volunteering once they become aware of the intervention nature of the study.

Participants will be remunerated via two payments of $100, one given at baseline assessment and one at the 6-month follow-up. It will be made clear to participants via the participant information sheet and verbally that they will be compensated for their contributions to the study and that the payment is given for them to use for any expenses incurred as a result of participating.

#### Sample/Randomization

In line with samples used in previous related research [29,31], 400 retired/non-employed individuals in good health aged 60+ years will be recruited into the study. A power calculation was carried out in G*Power to calculate the required sample size for power of 0.80 to detect any main effect or interaction at an alpha level of 0.01 and a conservative effect size of 0.1. A sample size of 370 (1:1 allocation) will be sufficient to detect a difference in effects between groups at post-intervention. These numbers take into account a loss of 25 % of participants during the course of the study. Allowing for potential effects of various extraneous influences, such as protocol adherence, 200 participants per group will be required (total n = 400).

After being screened for eligibility, half the sample will be randomly assigned to an intervention group (*n* = 200) and half to a control group (*n* = 200). The randomization process will be conducted using a computer-generated randomization script by a researcher who is blinded to the nature of the study and independent from participant recruitment and data collection/analysis processes.

#### Procedure

Consenting participants will be contacted to arrange a physical assessment and face-to-face interview. The assessments/interviews will be conducted on the campuses of two of the institutions involved in the study. These campuses are located 37 km apart and hence provide wide geographical coverage of the Perth metropolitan area.

Prior to arriving for their interview, participants will be asked to complete a battery of psychological and physical health status questionnaires. Sociodemographic information (age, gender, socioeconomic status, marital status, and ethnicity) will also be collected. Those participants with a preference for using the Internet and who have access to a computer will be asked to complete the questionnaires online. Participants without access to a computer will be posted out a paper version to be returned via a reply paid envelope or bring to their first interview. The instruments that will be used to collect psychological data from participants are listed in Table 2. The information and consent forms will be included at the front of the online survey for those participants using the internet, and paper copies of the information and consent forms will be included with the paper surveys that are posted out.

**Table 2:** Psychological measures used in the assessment protocol (primary and secondary)

Outcomes	Instruments
***Primary***	
Psychological Well-Being	Warwick-Edinburgh Mental Well-Being Scale [77]
Depression	Center for Epidemiologic Studies Depression Scale [78]
Self-esteem	Rosenberg Self-Esteem Survey [79]
Quality of Life	Global Quality of Life Scale [80]
***Secondary***	
Personal Growth	Personal Growth subscale of Ryff’s Psychological Well-Being Scales [81]
Purpose in Life	Purpose In Life subscale of Ryff’s Psychological Well-Being Scales [81]
Social Support	Social Provisions Scale [82]
Self-Efficacy	Generalized Self-Efficacy Scale [83]

At their face-to-face interview, participants will undertake a battery of tests to assess their physical health. These will include physical function tests (chest press, seated row, leg extension, chair rise to standing, 400 m walk, 6 m backward walk, 6 m normal pace walk, 6 m fast pace walk [60]), resting heart rate, blood pressure, height, weight, and waist girth. A qualified exercise physiologist will be present at all times, and any adverse events during testing will be managed appropriately and reported to the Ethics Committee as per University requirements. In addition, participants will be interviewed on their attitudes to volunteering and any perceived barriers, motivators, and facilitators relevant to commencing and maintaining volunteering behaviors. During an open-ended interview, participants will be asked about their history with volunteering to control for this potential confound, and their current and historical engagement in physical, mental, and social activity will be assessed to control for the degree to which isolation is mitigated by active memberships in recreational and/or social groups.

All participants will be asked to keep a daily record of their physical activity during the six-month study period, including the nature, duration, and perceived exertion of each physical activity episode. Those in the intervention condition will also be asked to keep a daily record of their volunteering activities, once again including details of the nature, duration, and perceived exertion of each episode.

Participants will be also asked to wear a pedometer and note the daily number of steps and engagement in activity in their activity diaries. Although pedometers are considered generally effective for measuring physical activity among older people [61], they are limited in their ability to measure and quantify the intensity of physical activity or detect periods of sitting or lying, which are critical for the evaluation of sedentary behaviors. All participants will therefore be asked to wear accelerometer-based activity monitors (GT3X+, Actigraph, Pensacola, FL) for 7 days after their first interview and for 7 days before their second interview to measure these outcomes.

The second interview will occur at the end of the six-month study period. Participants will be asked to complete the same battery of measures they completed at baseline. They will then participate in the follow up face-to-face assessment. This follow-up assessment will involve the same protocol as the first, with additional questions relating to volunteering experiences in the intervention group. These questions will include descriptions of the types of volunteering undertaken, their level of satisfaction with and enjoyment of their volunteering activities, and factors such as the level of recognition received and any skills they accrued [62]. A typology of volunteering activities will be developed and used to code responses [63,64]. Relevant barriers, motivators, and facilitators to initial and continuing participation will be assessed. Participants in the control group will also be asked to report on any participation in volunteering activities to assess for potential confounds. Should any adverse events be reported in this follow-up interview, they will be documented and submitted to the University Ethics Committee. Participants in both conditions will be provided with a report on their individual results. Physiological testing and interviewing processes will be managed to ensure that participants in the two groups (control vs volunteering intervention) do not interact with each other. The intervention and control conditions are described below.

#### Intervention

Those assigned to the intervention group will be required to undertake a minimum of 60 min of volunteer work per week. They will be advised that they can undertake any form of volunteering they wish during the study period, as long as it meets the definition of volunteering stated above. Participants will be provided with access to the services of a volunteering service organization to ensure they can select a position they find attractive and fulfilling. This will also assist in ensuring that participants are able to secure and commence their volunteering work in a timely fashion. As previous research has identified the importance of goal-setting in activity initiation [65], the purpose of the discussion with the volunteer organization representative will be to commence the participants’ decision-making processes in relation to the volunteering activity they will undertake. The volunteering representative will be available to provide ongoing support to all participants in the intervention condition throughout the study period. Participants will also have the option of contacting the study coordinators should they have any difficulty sourcing or maintaining volunteer work.

Having a choice of volunteering activity is important given the findings of previous research that flexibility is a key characteristic of successful volunteer programs [66].  Tang et al. [67], for instance, found that over three-quarters of seniors rated the choice of volunteer activities and setting their own schedule as being important. Numerous studies have also noted seniors’ desire to be engaged in pastimes that they find meaningful and rewarding [40,55,68,69]. As such, while seniors will have access to advice relating to potential volunteering opportunities in their local area, the final choice of activity will be theirs.

#### Control

Participants assigned to the control condition will be advised to maintain their existing lifestyles or take on new activities as they see fit. For ethical reasons, those with a sedentary lifestyle at baseline will not be required to maintain this lifestyle over the study period.

An attrition rate of 25 % is expected over the six-month intervention study [70,71]. Participant remuneration and responsive administrative processes (e.g., accessible, friendly staff and flexibility in interview scheduling) are expected to prevent excessive attrition. In addition, the participants will be asked to return their physical activity diaries on a monthly basis, which will facilitate follow-up contact with those who appear to be having difficulty or who may be at risk of withdrawing. The diaries will be user-friendly and require basic information in the form of steps per day, type of activity (e.g., swimming), duration (e.g., 30 min), and intensity (light, moderate, or vigorous). Example entries will be included in the diaries to demonstrate correct completion and to reduce the likelihood of respondent fatigue.

**Figure 1:**
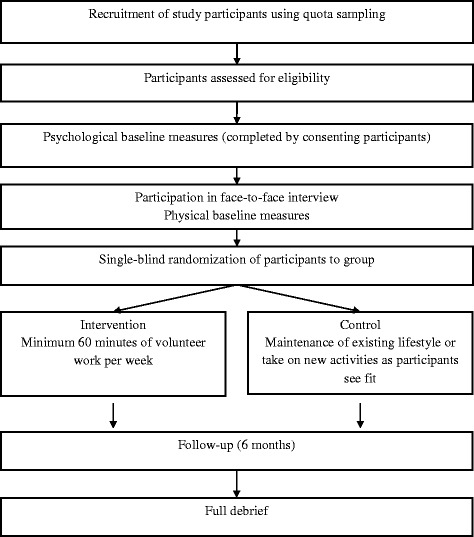
CONSORT diagram of the study design

The CONSORT diagram of the study design from recruitment to debrief is presented in Fig. 1.

### Outcomes

#### Primary

The primary physical outcomes (dependent variables) will be physical activity (steps taken; time spent in light, moderate, and vigorous physical activity; and sedentary time - measured using pedometers and activity monitors) and physical health (measured using a battery of physical functioning tests, resting heart rate, blood pressure, BMI, girth). The primary psychological outcomes (dependent variables) will be psychological well-being, depression, self-esteem, and quality of life (measured using the questionnaires listed in Table 2). Life satisfaction, assessed via a single-item, will also be a primary psychological outcome. Secondary outcomes will be self-reported personal growth, purpose in life, social support, and self-efficacy (measured using the questionnaires listed in Table 2), and attitudes to volunteering (assessed via open-ended interview and an amended version of the Community Service Attitudes Scale [72]).

### Analysis

#### Quantitative analyses

Analyses will be conducted using the intention-to-treat principle, based on all randomized participants. Despite randomization, baseline differences in groups may still occur due to chance. Similarity of baseline characteristics of intervention and control participants will therefore be assessed using appropriate descriptive statistics and inferential analyses (e.g., independent samples *t*-tests, Mann Whitney *U* tests, Pearson’s chi-square statistic, ANOVA). Baseline data will be also examined to analyze systematic bias in attrition.

##### Primary analyses

The primary analyses will utilize a between groups design with two time points (baseline and post-intervention) and two groups (volunteer vs. control). The primary independent variable is therefore condition. Relevant analyses (e.g., multivariate analyses of covariance) will be performed to explore pre to post differences in measures of physical and psychological health while controlling for baseline scores on those variables and any other relevant confounders (e.g., control group members engaging in increased physical activity because recommended to do so by their medical practitioners). An alpha level of *p* < .05 will be the criterion for significance in all statistical comparisons. Estimates of effect size will also be calculated.

##### Secondary analyses

Secondary analyses will utilize a within subjects design to assess for differences within groups from baseline to follow-up. Relevant analyses (e.g., paired-samples *t*-test) will be conducted to assess for these within-group differences.

All available practical steps will be taken to avoid missing data. Potential implications of missing data will be explored by using multiple imputation techniques. Baseline scores of participants who were successfully followed up will be compared with the baseline scores of those who withdrew to test for any systematic bias that may have been introduced through attrition.

#### Qualitative data

The interview data will be transcribed and coded using NVivo software to facilitate analysis of the factors identified by participants as affecting their attitudes and behaviors relating to volunteering activities. Initially, a deductive coding schema will be generated from the relevant literature, including behavioral models such as the Health Belief Model [73] and the Theory of Planned Behavior [74]. This coding schema will be inductively updated with emergent codes as data analysis progresses to allow early findings to guide subsequent data collection episodes [75,76]. As new codes emerge, earlier data will be re-coded to ensure coverage of all relevant themes. NVivo’s sophisticated search functions will facilitate this process. Data analysis will occur via the interrogation of individual content nodes (nodes being the storage points for content assigned to specific codes), by conducting text and matrix searches, and by reviewing the entire transcripts. This process will yield a comprehensive account of the relevant barriers, motivators, and facilitators relevant to seniors’ participation in volunteering activities. Among the intervention group participants, any changes in attitudes to volunteering over time will be documented.

Data storage will be managed as per the Curtin University Ethics Committee requirements. Electronic data will be stored on secure data servers and hard copy materials will be retained in locked filing cabinets.

## Discussion

This paper provides a comprehensive description of the methodology used to implement and evaluate a volunteering intervention for seniors. If successful, the trial will generate methodologically sound results that provide knowledge relating to the physical and psychological health benefits of volunteering by seniors. This information is needed to inform the development of public policy and interventions that have the potential to improve the lives of older Australians.

## Trial status

Ongoing

## Abbreviations

ABS: Australian Bureau of Statistics
ARC: Australian Research Council
WA: Western Australia
